# Accessibility of ART in the farming community of OR Tambo District Municipality in the Eastern Cape province

**DOI:** 10.4102/hsag.v29i0.2701

**Published:** 2024-12-18

**Authors:** Lorraine N. Mntonintshi-Mketo, Phillip Nhlanhla

**Affiliations:** 1 Department of Sociology, Faculty of Human Sciences, University of South Africa, Pretoria, South Africa

**Keywords:** accessibility, anti-retroviral treatment, exploring, farming community, human immunodeficiency virus, farm worker, OR Tambo District

## Abstract

**Background:**

South Africa accounts for 14% of all new HIV infections representing the highest annual rate of new HIV infections globally. In addition, South Africa is home to 21% of the worldwide HIV burden, with 7.97 million people living with HIV. HIV not only affects the health of those living with the virus but also impacts their economic well-being.

**Aim:**

The study aimed to develop an in-depth understanding of the accessibility of anti-retroviral treatment in the farming communities of the OR Tambo District Municipality in the Eastern Cape province.

**Setting:**

The study was conducted in two local municipalities of OR Tambo District Municipality in the Eastern Cape province.

**Methods:**

A qualitative approach was used to explore and describe the accessibility of anti-retroviral treatment (ART) in the farming communities.

**Results:**

The study revealed that travelling long distances, transport costs, lack of transportation and traditional beliefs were the major barriers for farming communities to access ART.

**Conclusion:**

The findings of the study propose that poor access to ART in farming communities is linked to socio-cultural status, weak social support and limiting socioeconomic status.

**Contribution:**

The Eastern Cape Provincial Department of Health’s management can use the study’s findings for recommendations to the National Department of Health management on how to improve HIV roll-out initiatives.

## Background

The availability of antiretroviral treatment (ART) has enabled people living with HIV (PLHIV) to live long productive lives. In South Africa by the end of 2022, at least 90% of PLHIV were on ART, which is an improvement from 70.7% who were on ART in 2017 (Human Sciences Research Council [HSRC] 2023). Although progress has been made in increasing the number of PLHIV on ART, disparities in ART coverage still exist. In South Africa, access to treatment is lowest among farming communities, which is where this study was conducted (Frescura et al. 2022). Several factors are attributed to this low coverage, which includes personal, healthcare system and social factors.

The global epidemic of HIV and AIDS remains a concern because of disparities in accessing ART. It is essential to manage HIV infection carefully using ART (Kemnic & Gulick 2022). Antiretroviral treatment denotes all medications used to stop HIV from replicating (Thompson, Aberg & Hoy 2012). Antiretroviral treatment is a combination of three or more antiretroviral medications used for inhibiting viral replication to prevent and treat HIV infection (National Agency for Control of AIDS 2019). An antiretroviral drug (ARV) is a drug used to lower the viral load in PLHIV (Van, Tlou & Dyk 2017). Frescura et al. (2022) added that with adequate ART, people living with HIV can live an average of 33 years.

The World Health Organization (WHO 2019) states that there is a possibility to eliminate the HIV epidemic as a public health emergency by 2030 with extensive global scaling up of HIV prevention, care and treatment. To achieve this, the Joint United Nations Programme on AIDS (UNAIDS) set targets in 2020 for HIV elimination on a continuum of care by outlining the 95-95-95 targets to be achieved in 2025 (WHO 2019). The first ‘95’ refers to 95% of people who are tested for HIV and know their status, the second ‘95’ means 95% of people who know their status are on ART and the last ‘95’ denotes that 95% of people on ART are virally suppressed (Frescura et al. 2022). These targets were built from the UNAIDS 90-90-90 targets that were set in 2015 and were supposed to have been attained in 2020 by the UNAIDS (Frescura et al. 2022).

One of the main issues affecting ART roll-out for the attainment of the 95-95-95 targets is accessibility. Coupled with immediate ART initiation, universal access to ART forms the standard for HIV treatment and prevention (Myburgh et al. 2021). Universal access to HIV treatment refers to the availability of ART, in terms of affordability, acceptability and reachability (Myburgh et al. 2021). The need to increase access to ART can be traced back to the WHO’s first ART accessibility initiative ‘3 by 5’, which sought to ensure three million people on ART by 2005 (Haire 2020). The ‘3 by 5’ initiative was implemented from 2003 to 2005 enabling the access of ART to low-to-middle-income countries (LMIC) (Haire 2020). Financial support for ART programmes in these LMICs is from the Global Fund on HIV and the United States President Emergency Fund on HIV and AIDS (PEPFAR) since 2003 (Haire 2020). The financing of ART programmes in LMICs has enabled the provision of free ART in LMICs including South Africa that initiated its ART rollout programme in 2004 (Lilian et al. 2020). South Africa manages the world’s largest ART programme with at least 4.6 million people having accessed ART by the end of 2018 (Burger, Burger & Van Doorslaer 2022).

Although South Africa has the largest ART programme and has made significant improvements since 2004 to increase ART uptake, the country compares poorly to other African countries (Mnyaka et al. 2021). For example, in Rwanda, HIV-related deaths declined by 76% between 2010 and 2014, which indicates effective ART use (Mnyaka et al. 2021). In addition, South Africa also performed poorly in achieving the UNAIDS 90-90-90 targets by the end of 2020, managing to achieve 71% of PLHIV on ART. According to Mnyaka et al. (2021), 95-95-95 targets have also not been attained, necessitating aggressive action towards universal access to ART especially in rural areas where ART access has been inadequate. In the Eastern Cape, where the OR Tambo District Municipality is located, HIV and AIDS-related illnesses are the leading cause of death (Mnyaka et al. 2021). This study helped to illustrate ART accessibility in the farming communities of the OR Tambo District Municipality.

### Aim of the study

The study intended to develop an in-depth understanding of the accessibility of ART in the farming communities of OR Tambo District Municipality in the Eastern Cape province.

## Research methods and design

### Study design

Saunders, Lewis and Thornhill (2019) assert that the research design is the plan that determines how the research questions will be answered. Exploratory and descriptive research designs were used to explore and describe the study’s aim. The choice of research design and data collection method that were utilised in this study was based on the research objectives intended to understand the accessibility of anti-retroviral treatment in the farming communities.

### Setting

The study was conducted in the identified wards in two local municipalities of the identified district in the Eastern Cape province.

### Study population and sampling strategy

The targeted population in this research study were OR Tambo District Municipality farm workers and farm owners of any gender above 18 years of age in the farming communities.

Non-probability convenient purposive sampling methods were used to choose the study sample. For this research study, any volunteer who was willing to participate while meeting the inclusion criteria was purposively chosen by the researcher. Conveniently, the researcher interviewed the participants who were on duty and willing to participate. The researcher recruited and sampled the participants at two farming communities, one from Nyandeni local municipality. The researcher recruited 25 participants, 10 farm workers with 2 farm owners from Nyandeni Local Municipality and another 10 farm workers with 3 farm owners from Port St. Johns Local Municipality.

The following inclusion and exclusion criteria were followed:

In this regard, the criteria for inclusion in the final sample were as follows:

- Women and men older than 18 years regardless of their HIV status.
- Farm workers living and working in the farming community of OR Tambo District Municipality, irrespective of their gender.
- Farm owners of the identified farms in the OR Tambo District.
- Farm workers and farm owners willing to participate in the study without compensation.
- Farm workers and farm owners who consented to participate and have the interview audio recorded.

Meanwhile, the criteria for exclusion in this study were:

- Women and men below the age of 18 years.
- Non-resident and working in the identified farms.
- Farm workers and farm owners who cannot give consent.

Before gathering data, the researcher made sure the participants understood the goal of the study. Before giving their agreement to participate in the study, the participants were fully informed about the risks, benefits and their rights. Every participant had the option to agree or reject taking part in the research project. Before starting a semi-structured interview, researchers obtained participants’ completed informed consent forms and explained the goal of the study.

### Data collection methods and procedures

The researcher used semi-structured interviews in this study in addition to gathering secondary data by doing a literature review. In order to maintain control over the interview process by enabling participants to provide more detail about their answers to the interview questions, the interview guide was developed ahead of schedule (Saunders et al. 2019). Every interview began with the same general central question: ‘how do people in the farming communities of OR Tambo District Municipality access ART?’

### Data collection

An initial pilot study was conducted before the actual data collection to ascertain whether the interview schedule could successfully acquire the required data from the participants. The pilot study involved four research participants who met the criteria for sample inclusion. Two more persons were removed from the research project after that. In-depth semi-structured interviews with 25 participants were used by the researcher to gather data. Twelve participants at one of the selected farms in the Nyandeni local municipality were interviewed by the researcher between 10 September 2023 and 30 September 2023, in order to gather data initially. Interviews were conducted with 13 additional participants from the other two selected farms in the Port St. Johns local municipality between 01 October 2023 and 25 October 2023. An in-depth individual interview was conducted with the aid of an interview guide. The method the researcher employed was called qualitative observation, which involved making field notes about the individuals’ actions and nonverbal cues. However, there were no difficulties in gathering the data, and saturation was never achieved.

Prior to analysis, all transcripts – including audio recordings, notepad notes and copies of consent forms – had been anonymised. The researcher utilised a tape recorder to ensure she did not miss any information provided by the interviewee. The interviewee was given a consent form to sign prior to the start of the interview. The researcher fully explained everything to the interviewees in terms they could understand before signing the consent form. This indicates that the interview was conducted in the respondent’s native language.

### Data analysis

The procedure to analyse data was adapted from Braun and Clarke’s (2022) six steps of thematic analysis. Step one involved data familiarisation whereby the researcher listened to the audio recordings and read the notes captured in the notebook. In step two, the researcher created initial codes. In step three, the researcher searched for themes. These themes were developed from the information from the interviews. In step four, the researcher reviewed the themes using the objectives and research questions. Step five involved the researcher defining the themes and sub-themes. In the last step, a report was produced.

### Measures to ensure trustworthiness

Trustworthiness and authenticity in qualitative research are determined by four indicators and these are credibility, dependability, transferability and confirmability (Kumar 2019). Polit and Beck (2021) discuss the issue of trustworthiness in qualitative research and trace these four indicators of trustworthiness described by Kumar (2019).

#### Credibility

In qualitative research, credibility is synonymous with internal validity and describes how plausible the study’s conclusions are in the eyes of the participants (Kumar 2019). When study participants validate, confirm and endorse the study findings, the study has greater credibility (Kumar 2019). Long-term participation suggests that the investigator dedicated sufficient time to data collection activities to completely comprehend the accessibility of ART in rural regions. The researcher gave the study results to the participants for confirmation, validation and approval in order to guarantee reliable study results. During the investigation, the researcher additionally employed bracketing (reflexivity) to ensure that their own ideas and biases did not undermine the perspectives, experiences and attitudes of the research participants.

#### Transferability

Transferability, as defined by Kumar (2019), is the extent to which the findings of qualitative research may be extrapolated to different contexts or regions and is comparable to external validity in quantitative research. The researcher gave careful thought to field notes, observation and examination of non-verbal clues during the interviews. Furthermore, the description of the study environment is crucial for guaranteeing transferability because it clarifies the borders of the geographic area and the demographics of the population.

#### Confirmability

According to Johnson, Adkins and Chauvin (2020), achieving confirmability in research can be accomplished by reducing the impact of researcher bias on study findings and by providing an explanation of any researcher traits that may have an impact on those outcomes. In order to ensure confirmability, the investigator collaborated with skilled and knowledgeable researchers to verify that appropriate methodological procedures were followed in assessing the degree to which the study’s conclusions and findings were logically derived (Kumar 2019). Furthermore, the in-depth interview notes were scrutinised, and voice recordings were listened to guarantee that the data precisely represent the viewpoints of the participants. The study setup was described in order to guarantee transferability as well.

#### Dependability

Johnson et al. (2020) note that to ensure the dependability of study findings, researchers ought to provide a complete description of the methods and procedures used to conduct the study to enable other researchers to duplicate the findings. To enhance dependability, the analysed data were checked by a peer researcher who is a final year PhD candidate at the University of South Africa in the Health Department, as a means of ensuring findings are consistent with what the researcher had found. Dependability in the study was also ensured by testing the data collection instruments on four participants (one farm worker and one farm owner who did not form part of the final sample and one farm worker and one farm owner who formed part of the sample). This pre-testing of the data collection instruments provided the means of ensuring that there were no unambiguous questions.

### Ethical considerations

Ethical approval to conduct this study was received from the College of Human Sciences Research Ethics Review Committee, University of South Africa with ethical clearance number 43291589_CREC_CHS_202.

The method employed by the researcher was qualitative observation, which involved making field notes about the individuals’ actions and nonverbal cues. However, data saturation was never achieved. There were no difficulties in gathering the data. Before analysis, all transcriptions – including audio recordings, notepad notes and copies of consent forms – were anonymised. The researcher used a tape recorder to ensure that she did not miss any information provided by the interviewee. The interviewee was given a consent form to sign before the start of the interview.

## Results

### Biographical characteristics of the participants

#### Age categories of the participants

Most participants (40%) were aged 51–70 years. The second most represented age group was the 31–42-year age group.

#### Gender demographics of participants

There were 25 participants (*n* = 25, 100%), with the majority (*n* = 15, 60%) participants being females, while 10 (40%) participants were males. The researcher’s primary interest was to develop an in-depth understanding of the accessibility of ART in the farming communities of OR Tambo District Municipality in the Eastern Cape province. While gender was not necessarily the study’s area of main concern, the issue of gender was included largely to compare the gender that was mostly affected by the socioeconomic ramifications of HIV and AIDS. The ensuing section presents the marital status of the participants in the study. From the viewpoint of the researcher, the issue of marital status is critical. Among others, this variable illuminates the level of socioeconomic and related hardships faced by families.

#### Marital status of the participants (*N* = 25)

Most of the participants (*n* = 13, 52%) who participated in the study were married. There was an equal number (*n* = 6, 24%) of single participants and those who were widowed.

#### Educational levels of the participants

The level of education of the participants indicates that many of the participants (*n* = 11, 44%) had each completed Grade 10, Grade 11 or Grade 12. Another nine (36%) participants had each completed Grade 6, Grade 7 or Grade 9. There were only three participants (*n* = 3, 12%) who had tertiary education. There were two participants (*n* = 2, 8%) who only had completed Grade 5 or below. In this regard, the researcher opines that the participants are literate as most participants had high school education, which had the highest educational level cohort, followed by those participants who had primary education. This level of education could also have contributed to their abilities to respond with ease to the interview questions.

#### Home language demographics of participants

Most participants (*n* = 22, 88%) speak IsiXhosa, which is the dominant language spoken in the OR Tambo District Municipality. Only three (*n* = 3, 12%) participants had a home language that was different from the rest of the participants. Although the three participants’ home language was not IsiXhosa, they were able to speak isiXhosa and attributed this ability to having lived in the OR Tambo district for more than 3 years. The researcher conducted the interviews in IsiXhosa to enable participants to express themselves with ease. The interview guide was originally written in English and was translated into IsiXhosa for participants to understand terminology related to HIV and ART.

#### Other source of income of participants

The participants’ other source of income play a crucial role in assessing their financial capacity to meet the socioeconomic challenges they encounter. Income from sources other than their labour on the farms is included in this. Seven participants (*n* = 7, 28%) were not receiving any other source of income and solely depended on the remuneration from farm work, while another seven participants (*n* = 7, 28%) received income from the South African Social Security Agency (SASSA) child support grant. Five participants (*n* = 5, 20%) were receiving additional income from the SASSA old age grant. Only 4% (*n* = 1) of the participants received disability grants from the SASSA. Lastly, only five participants (*n* = 5, 20%) received alternative income from business ownership.

Data were collected from a sample of 25 participants. Three main themes and 12 sub-themes emerged from the data analysis.

## Themes and sub-themes

### Theme 1: Difference between HIV and AIDS

Numerous descriptions emerged concerning what participants understood as the difference between HIV and AIDS. However, these responses illustrated that participants knew the difference. In their explanations, participants described HIV as a virus and AIDS as a disease. For instance, some participant farm workers and participant farm owners shared their differences as follows:

‘HIV is when you are sick but not entirely sick. Some people find out they are positive by feeling sick. AIDS is beyond HIV, meaning that it is when you are very sick and almost bedridden. It is when your immune system is much compromised to the extent that chances of surviving are slim.’ (Participant 1, female, 59 years old)

‘HIV is a virus mam; it attacks the immune system of the HIV-negative person. It can remain in the body for many years not knowing you are infected with it. That is why there are constant reminders for people to test for HIV so that they know about their HIV status. AIDS happens when the person is sick with many diseases due to not taking ART.’ (Participant 18, female, 61 years old)

‘HIV is a virus and AIDS is a disease mam.’ (Participant 19, male, 32 years old)

‘According to my understanding, HIV is an incurable disease or virus which is killing anyone who does not take responsibility for prevention. HIV is different from AIDS because it is a dying stage of a person not taking treatment. AIDS is scarier than HIV. Once you develop AIDS chances of recovering are few.’ (Participant 21, male, 55 years old)

### Theme 2: Anti-retroviral treatment knowledge in the farming community

The study explored participants’ knowledge of ART. Most participant farm workers and three participant farm owners noted that ART is the medication that suppresses and prevents HIV from spreading in the blood and preventing HIV’s further transmission. Knowledge of ARVs assists people in understanding the importance of treatment adherence. In this regard, participants were quite knowledgeable about ARVs including those who were taking them. In their exhibit of knowledge, participants explained, how ART works, that it was a lifelong treatment, and delineated the benefits of adhering and the consequences for not adhering. Furthermore, some responses detailed how ARVs work; this included a description of ARVs, reducing the viral load and the consequent effects of HIV on CD4 cell count. The participants responded as follows:

‘ARVs are pills that are provided by the Department of Health clinics to suppress the progression of it spreading. ART is important for the survival of life, for instance, my sister was diagnosed with HIV in 1996 and survived on pills which were too many but were reduced to one pill in 2008. If you take the ARVs the correct way, you are more likely to live long.’ (Participant 1, female, 59 years old)

‘ART are different kinds of HIV management treatment. For instance, ARVs, nevirapine, pills that prevent HIV infection after sleeping with the person whom you might think is HIV positive.’ (Participant 8, female, 57 years old)

‘It is a treatment used for managing HIV from spreading. It is a control measure introduced by the government.’ (Participant 9, female, 51 years old)

‘When we visit the clinics, we are told by doctors and nurses ART are ARVs that suppress the viral load in the body and if not taken properly the virus will go up and you can contract other diseases.’ (Participant 14, male, 32 years old)

### Theme 3: Experiences in accessing anti-retroviral treatment in the farming communities

The participant farm workers shared their experiences of having difficulties accessing ART and other chronic medications at the local clinics. Sub-themes emerged from their explanations of experiences of stigma and discrimination and other structural problems such as lack of transport to clinics, long queues, overcrowding and negative nurses’ attitudes when being attended.

#### Sub-theme 3.1: Stigma and discrimination

Participant farm workers shared that it was difficult to access ART at the clinics because of stigma and discrimination. In their shared experiences, participants revealed that fellow community members who collected medication for other chronic illnesses except HIV discriminated against them. This sub-theme was developed from the following quotes:

‘There is a lot of stigma and discrimination around HIV. People still regard HIV as a disease that is meant for dogs and once you have it you are like a dog. As a result, some people fear being seen in clinic queuing where nurses are rolling out the ART. Some end up stopping taking the treatment fearing being seen or known to be collecting ARV pills.’ (Participant 1, female, 59 years old)

‘As for me, I feel like people who are there in the clinics to collect ART or ARVs are stigmatised and discriminated against because there is a clear description of written sections for the collection of ARVs. Another thing is that sometimes people taking ART experience dizziness, tiredness, nausea, and burning sensation in their chest as a result they stop fetching the treatment in their clinics.’ (Participant 17, female, 44 years old)

‘I am not aware of any difficulties since I am not living with HIV nor do I have a friend or relative who is living with HIV. But what I have noticed is that in the clinics people living with HIV have their section for a collection of medication, don’t you think that is discrimination with the stigma attached? How do clinics expect ARVs collectors to not miss their collections and reviews when they are being exposed like that?’ (Participant 2, male, 39 years old)

#### Sub-theme 3.2: Structural problems in accessing anti-retroviral treatment

Most participant farm workers reported that their problem was referral to other clinics to collect ART or medication for other chronic illnesses because of stock outs. Other participants shared that inflexible clinic appointment dates were problematic considering that they would also have farm work commitments. From these shared experiences, participants noted that their bosses in the farms found it difficult to understand such rigid clinic appointments. Other issues discussed by the participants were the long waiting times they experienced because their clinic files were missing, and they would wait while a new file was being created. The participants further shared that there is overcrowding in clinics, which results in long queues. Long-distance and transport issues were reported by the participants, and this sometimes resulted in missing clinic appointments. Easy access to ART centres and affordable transport costs promote adherence. The following quotations illustrate the structural issues shared by the participants:

‘Honestly speaking, the health facility that I am going to when there is a need for more rooms or offices for consultations, is very much overwhelmed. The number of ART clients does not match the size of the health facility.’ (Participant 20, female, 66 years old)

#### Sub-theme 3.3: Nurse attitudes

Some participant farm workers shared their experiences regarding the negative attitudes of nurses towards patients. Such negative attitudes create poor interpersonal relationships between clients and healthcare providers, resulting in limited ART access and non-adherence. The following are the responses by the participants:

‘The nurses can pose negative attitudes when hesitating to continue with the ART because sometimes it is hard to take treatment [*ART*] on an empty stomach. It’s easy if there is food, this could be one of the reasons why people miss taking ARVs from clinics.’ (Participant 15, female, 27 years old)

‘The only thing is administration in terms of the information and some negative attitude from the staff members.’ (Participant 14, male, 32 years old)

#### Sub-theme 3.4: Social support affects access to anti-retroviral treatment

Participant farm workers described how social support affected their access to ART. This social support was from employers, family, friends and relatives. On the other hand, farm owners revealed that they were supportive of their employees accessing ART. Such support included inviting healthcare workers to their farms and allowing days off work for those who have clinic appointments. Other participant farm owners responded that they occasionally get healthcare workers to render services to the farm workers. The participants’ responses regarding their employee access to ART were as follows:

‘Not having sufficient support from close family or friends and relatives can cause a person to stop taking ARVs or consult clinics for medical issues. Even at work if you are not shown support by the employer you can end up stopping taking ARVs from the clinics fearing of losing the job if constantly asking for time or day offs.’ (Participant 9, female, 51 years old)

### Theme 4: Healthcare visits to the farms

The participants were asked how often healthcare workers from the clinics visit their farms for service provision. Most participant farm workers responded that there has never been any visit by healthcare workers to a farm workplace. The following quotes illustrate the development of Theme 4:

‘At the place I live, there are no such visits. The only visit by health care providers was way back in 1997 when the virus was at its peak and people were dying and those who were very sick refused to get tested.’ (Participant 1, female, 59 years old)

‘At my workplace, there have never been any health care providers visiting. We as workers once requested that there should be wellness days whereby healthcare workers visit us for health education. But our employer always promises to follow it up till today.’ (Participant 6, female, 70 years old)

‘Since working here, there has been no visit by nurses or caregivers.’ (Participant 10, female, 36 years old)

### Theme 5: Access to HIV and AIDS information

The participant farm workers were asked how they access information about HIV and AIDS. Many participant farm workers revealed that they accessed HIV and AIDS information from social media and mainstream media sources such as newspapers, television, radio and Facebook^®^, while other participants reported not having access to HIV and AIDS information. The following quotes support this theme:

‘I access HIV and AIDS information through social media, for instance, Facebook®, television, and radio. I can access all these on my phone because most of the time I work and arrive at my rented room tired then just use my smartphone to access each platform.’ (Participant 3, male, 38 years old)

‘I access information through radio, television, and visits to the clinic when I collect my ARV pills. But I am due to collect them in the pharmacy, but it is too far, so I rejected the offer.’ (Participant 4, female, 42 years old)

‘Following Facebook^®^ pages and YouTube^®^ is how I access accurate information about ART and HIV.’ (Participant 7, female, 27 years old)

‘For me, I cannot only access HIV information when I visit the clinic for reviews or for picking up my HIV pills. We are provided with different kinds of information there in pamphlets and occasional presentations by nurses.’ (Participant 6, female, 70 years old)

### Theme 6: Barriers to accessing anti-retroviral treatment in farming communities

The participant farm workers were asked to explain barriers that are or might be preventing PLHIV from accessing ART. The participants outlined various barriers such as long distances to the clinics, stigma, discrimination-related concerns and a lack of health care visits to their farms. In addition, the participants noted the lack of confidentiality and privacy, lack of HIV and AIDS awareness and information, long queues, negative healthcare worker attitudes, poor knowledge of drug regimens, fear of side effects and religious or traditional beliefs about ART also hindered ART access.

#### Sub-theme 6.1: Distance to the clinics

Most participant farm workers and some participant farm owners described the issue of long distances to clinics. In their descriptions, the participants explained that they travelled long distances to clinics to access their ART, other chronic medications or even basic healthcare needs. Noteworthy, the issue of long distance to the clinics was described by participants from all five wards where data were collected in the two local municipalities. This sub-theme was supported by the following quotes:

‘I am far away from the clinic. As you can see our farm is in deep rural areas. I am sure you struggled to get here. The road is rough with gravel. There is only one bus we use for transport to go to town and the clinic. When you travel by foot to the clinic it is almost 50 km. You can never make it back to work or home by foot, you must find a nearby place and ask for a place to sleep.’ (Participant 1, female, 59 years old)

‘When travelling by transport from my work, it takes almost two hours to reach the clinic. But when walking on foot it’s almost four hours and it is very hard to go back on foot again, but some people have to because of money problems because it costs about R60.00 for a return trip using transport.’ (Participant 4, female, 42 years old)

#### Sub-theme 6.2: Limited HIV, AIDS and anti-retroviral treatment awareness campaigns

Some participant farm workers shared that they knew of communities in other local municipalities that receive HIV, AIDS and ART awareness services yet they had never received any awareness campaigns in their municipality. From this comparison with other local municipalities, the farming community perceived that they had limited exposure to HIV, AIDS and ART awareness. The following quotes support the sub-theme:

‘I hear there are awareness campaigns that are provided by the clinics and Department of Health, but in this farm, I am working at we have not received any awareness campaign. It was only in 1997 whereby there was a visit by healthcare providers, that I can say maybe it was some sort of an awareness campaign.’ (Participant 1, female, 59 years old)

‘Maybe if we were to be educated on ART importance by the health department it would be easy to know our rights on basic health care services that we deserve as employees. The information I received from the Department of Health was three years back during the COVID-19 outbreak.’ (Participant 2, male, 39 years old)

#### Sub-theme 6.3: Beliefs regarding anti-retroviral treatment

Most participant farm workers shared that barriers to accessing ART may be the result of misleading beliefs about ART. Some of the participants shared that beliefs impeding people from accessing ART were associated with religion, while some beliefs were associated with witchcraft. In addition, some participants highlighted that ART access is hindered by some myths associated with taking ART that manifest as nightmares and that ART burns one’s liver. The following quotes support this sub-theme:

‘Once I attended a church which I cannot mention by name, in that church, it was forbidden to use treatment of any kind while being a member of that church. Even my sister who is living with HIV defaulted and became very sick to the point that she developed TB and was almost bedridden. She struggled to get back to health.’ (Participant 1, female, 59 years old).

‘I believe this disease was brought by people from outside our country. Others believe in these born-again churches; these strange churches promise us that they can cure this disease. I cannot believe that I prefer to continue using this ARV pill. Here I am, having tested positive 10 years ago, I am healthy, and nothing is stopping me from living my life.’ (Participant 6, female, 70 years old)

‘I have heard that HIV is a virus from witchcraft and there is no such thing as being infected. I also believe that it is a curse, or a disease used in witchcraft to destroy hardworking people. As for me, I will believe there is HIV when I test HIV positive myself. I have never used a condom but here I am, I do not feel sick at all except for minor fevers from the cold weather.’ (Participant 5, male, 23 years old)

‘There is one traditional doctor who after seeing my friend advised him to leave the medicines because his problem was man-made and not AIDS related. He told him that his skin problem was curable and that it was not AIDS. He told him to stop taking ARVs which he did although he was confused. He was convinced so he stopped taking ARVs for almost a year until he fell ill and had to go back to the clinic. He has since started the treatment again.’ (Participant 10, female, 36 years old)

### Theme 7: Measures to improve accessibility of anti-retroviral treatment among people living with HIV in the farming communities

The participant farm workers were asked if they had any suggestions to improve ART accessibility. From their responses, one main theme emerged, measures to improve ART access; this theme was supported by five sub-themes. Recommendations from the participants included the provision of educational awareness campaigns, training of healthcare staff on customer care services and distribution of chronic medication by healthcare givers to communities. The participants also recommended the use of mobile clinics especially for injectable ARVs. The participant farm owners responded to their wish to see things done differently in their farms regarding ART care. The farm owners further responded that they would like their farming communities to be assisted with transport to health facilities, the provision of mobile clinics and delivery of chronic medication to the community members especially their employees.

#### Sub-theme 7.1: Adequate information, education and counselling

Some participant farm workers highlighted the need for educational awareness campaigns on ART. The following quotes support this sub-theme:

‘I want HIV and ART awareness campaigns these should be done in our workplace and my community.’ (Participant 9, female, 51 years old)

‘I wish for constant visits by the Department of Health and the Department of Social Development to do awareness campaigns on HIV, AIDS, and ART. There is a great need for other services to be provided by these departments because some of my employees now qualify for the old age grant but cannot receive it because they do not have identity documents (IDs).’ (Participant 23, female, 59 years old)

‘I want HIV and other chronic diseases awareness campaigns to be done at my workplace. Our employer is denying access to such services complaining that he will be losing produce and money while spending time of the day attending to health care issues.’ (Participant 16, male, 46 years old)

#### Sub-theme 7.2: Positive relationship with health workers

Most participants shared that they have negative relationships with the nurses who attend to them. The participants perceived that the nursing care they received lacked compassion. The following quotes support the sub-theme:

‘I want health care workers especially those who deal with the ART rollout in the clinics to be trained on how to communicate with patients with respect. This is because when you get into the waiting room, they start calling you out aloud. We feel embarrassed because everyone can hear your name. There is a lack of confidentiality. This may be okay for those who are strong and do not self-stigmatise. But for me, I feel like not coming back.’ (Participant 12, female, 41 years old)

‘There is a need to add more consultation rooms at the clinic I usually go to. The nurses can be rude at times. They shout at waiting patients as though it is a show of authority and there is a lack of confidentiality.’ (Participant 19, male, 32 years old)

#### Sub-theme 7.3: Decentralising anti-retroviral treatment services outside of healthcare facilities

The participant farm workers and participant farm owners recommended that the government should distribute ART through community caregivers who should conduct home visits delivering the ART. The following quotes support the sub-theme:

‘I would like the government to bring the ARVs to our homes as home deliveries. There should be caregivers delivering all sorts of chronic medication to deserving individuals.’ (Participant 6, female, 70 years old)

‘The Department of Health must have delivery points of chronic medication, especially the HIV medications.’ (Participant 24, male, 60 years old)

#### Sub-theme 7.4: Injectable antiretroviral drugs

Some participant farm workers recommended that the Department of Health should introduce injectable ARVs that work for a fixed period. The following quotes support the sub-theme:

‘The Department of Health should now introduce once in six months injections as ARVs. These pills are a threat to our lives because even at home once it is known you are taking ARVs, the kids who smoke “Nyaope” break in to steal the pills. It’s frustrating really.’ (Participant 20, female, 66 years old)

‘If there is an injection, most people with HIV would prefer it. They should give a once-off injection.’ (Participant 10, female, 36 years old)

#### Sub-theme 7.5: Transportation and mobile clinics

Most participant farm workers and participant farm owners suggested that there should be an improvement in transport services to and from clinics and to town. The following quotes support the sub-theme:

‘There should be free transport like school buses so that we can travel to towns and clinics to get medicines.’ (Participant 18, female, 61 years old)

‘Department of Transport should provide our community with buses that we will not be required to pay to be transported to clinics and towns.’ (Participant 8, female, 57 years old)

## Discussion

Regarding the participants’ knowledge of the difference between HIV and AIDS, the majority of participants explained that HIV is controlled by ART and once you stop taking the medication you develop AIDS. On the other hand, some farm workers and farm owners highlighted that they did not know the difference between HIV and AIDS, rather it was the same thing. Some other participant farm workers shared that the difference between HIV and AIDS related to their experiences with HIV infection. The participants’ responses are supported by Hardy (2019) who states that AIDS is a disease caused by HIV. Human immunodeficiency virus infections do not always advance to Stage 3, which is the commencement of AIDS and several PLHIV live for years without getting AIDS (Hardy 2019). Hardy (2019) further states that a person living with HIV can anticipate leading a normal life because of advancements in treatment. Although an individual might live with HIV without having AIDS, HIV is present in all cases of an AIDS diagnosis. Even if AIDS never manifests, HIV infection never goes away because the treatment cannot cure the disease, but treatment is used to manage the disease.

Most participant farm workers and participant farm employers indicated that they knew ART as a medication that suppresses and manages HIV from spreading in the blood and preventing further transmission. Other participant farm workers and participant farm owners highlighted that ART are various HIV medications to prevent infection and prevent spreading after infection. Some participant farm workers responded that ART consists of pills provided by clinics and doctors to combat the spread of HIV and are not available over the counter. WHO (WHO & Joint United Nations Programme on HIV/AIDS 2022) states that prompt access to ART is essential for both improving the health of PLHIV and preventing HIV transmission. After testing positive for HIV people ought to start taking ART (WHO 2022). Furthermore, the UNAIDS 95-95-95 targets state that 95% of people are aware of their status, 95% of PLHIV are on ART and 95% of PLHIV on ART are virally suppressed. The WHO (2021) advises initiating treatment as soon as an individual tests positive for HIV. Anti-retroviral treatment initiation should be commenced immediately after an HIV-positive result regardless of whether a person is feeling well at that point. Findings from longitudinal studies concluded that those who begin ARV medication early tend to be healthier and have longer lifespans.

The participant farm workers were asked about their experiences in accessing ART. The participant farm workers shared different experiences regarding ART access. These experiences included stigma and discrimination, structural problems and nurse attitudes. Participant farm workers also shared their experiences of difficulties such as clinics running out of ARVs and other chronic medications, transport problems, lack of privacy during consultations, inflexible clinic schedules, long waiting times and inadequate space for consultations with consequent overcrowding. Based on these participant responses, there are infrastructural challenges that in turn affect experiences in accessing ART. In addition, participants were negatively impacted by the long waiting times especially at the reception area before they could access files and receive directions to the wellness section. The waiting at the clinics was further compounded by nurses who would take breaks for lunch and tea while patients waited for health service. This lack of timeous service delivery affects the overall health service delivery also resulting in negative experiences in accessing ART in farming communities. The participant’s responses are supported by Vitalis (2021) who stated that the clinic’s employee deficit and misplaced client files were highlighted as two of the causes of the lengthy wait times.

Participants were asked how often healthcare workers from clinics visit their farms for service provision. Many participant farm workers responded that there had never been any visit by healthcare workers. A minority participant farm worker revealed that there had been visits 3 years ago for COVID-19 vaccination and in 2022 for breast cancer awareness; however, none of these visits were for HIV, AIDS and ART-related services. WHO (2021) states that accurately identifying community members who have an impact on health, participating in community-based health initiatives and addressing the government’s obligations on health and equity are all important components of enhancing health literacy. Sustainable development goal 3 stipulates that increasing health awareness and health literacy programmes are vital components in ensuring good health and well-being. Furthermore, the Department of Social Development can also play a role in conducting HIV, AIDS and ART awareness campaigns through the use of NPOs they fund, which operate in rural farming communities.

When the participant farm workers were asked how they accessed HIV and AIDS information, nine participant farm workers noted they accessed information from social media. Okan et al. (2019) indicate that social media and health literacy are interconnected, especially in health promotion and prevention. Online and social media can be a cost-effective manner of reaching targeted audiences. Free Wi-Fi at drop-in centres and other farming community points should provide opportunities for access and use of social media to disseminate health information (Roberts, Callahan & O’Leary 2017). Few participant farm workers responded that they had no time to access information about HIV and AIDS, while the majority of participants accessed information by communicating with co-workers. It was evident from these participants’ responses that work commitments took precedence over seeking HIV, AIDS and ART information; therefore, it is reasonable for employers to set aside time for all employees to attend wellness programmes in the workplace. The purpose of the HIV and ART awareness campaign, according to Amrahs (2024), is to raise community awareness of issues of HIV and its prevention. The awareness provides information on HIV-related stigma, treatment accessibility, prevention and modes of transmission (Amrahs 2024). There are various ways to define awareness, and the terms are sometimes used synonymously with knowledge. Employee awareness indicates that they are learning more about the issue.

The participants outlined several barriers to accessing ART that included long distances to the clinics, stigma-related concerns, lack of healthcare visits to farms, lack of privacy and lack of HIV and AIDS awareness campaigns. Other barriers listed by participants included limited access to information, negative healthcare worker attitudes, poor knowledge of drug regimens and fear of side effects. The participants also described the issue of religious or traditional beliefs about ART impeding ART access. Moreover, being asymptomatic at the time of HIV diagnosis was also considered a barrier to accessing ART as PLHIV felt they were too healthy to start ART. With all the aforementioned challenges, the majority of participant farm workers explained the unavailability of transport resulting them in walking long distances. If transport was available, participants noted that it was unaffordable. As a result, of the time taken walking to clinics, participants would be unable to return to work and therefore take an unpaid day off from work. The participants’ responses are supported by Anthes (2019) who states that providing high-quality healthcare in rural settings is a global challenge. For people living in rural areas, long distances, geographical barriers, poor infrastructure and the high cost of transport affect routine medication access, patient utilisation of care and the level and capacity of healthcare workers at clinics and hospitals. McCormick, Osei-Anto and Martinez (2021) add that lack of access to services and poor retention in medical care in farming communities has been shown to predict poor health outcomes for PLHIV, most notably with lower white blood cells and more rapid disease progression. The participant farm owners also in concurrence revealed that they travel long distances to access healthcare. The farm owners further highlighted that while the employees spend most of their time on the road and in the clinics, they are losing productivity because of no work. Although this is more common in rural than in urban regions, MacKian and Simons (2020) claim that transportation costs and geographic distance to the clinics are impediments to ART care. Obstacles associated with clinics included lengthy waiting periods for medication collection and clinical reviews, overcrowding, negative attitudes from healthcare professionals and a shortage of health workers (MacKian & Simons 2020). There ought to be innovative ways to reduce obstacles in accessing ART as a growing number of PLHIV need to start treatment and stay on it for the rest of their lives. Other participant farm workers responded that there was a nearby clinic, but they chose to receive services from other clinics, which are far away because they feared their HIV status would be known in their workplace or community. Social obstacles like discrimination and stigma and a lack of personal or social support made it difficult for patients to stay in care and follow their treatment plans. When receiving HIV services, PLHIV was afraid that people they knew would see them at the clinic. Barrett, Ortmann and Larson (2022) further add that this fear is extended to delays in drug collection, involuntary disclosure during service delivery and a lack of privacy, which makes patients travel to different medical facilities outside of their community to receive care (MacKian & Simons 2020).

When participant farm workers and farm owners were asked about possible solutions to improve ART access, the majority of participants suggested solutions to mitigate the long distances to reach the clinics with the associated high transportation costs as well as addressing the long waiting times. Concerning the issue of transport, the participants listed possible solutions such as a community bus to be provided by the Department of Transport, mobile clinic provision every 2 months and the facilitation of wellness workshops by farm employers. Participants also suggested educational training for nurses on how to communicate and treat clients visiting the clinics, provision of a monthly supply of ARVs at each visit, provision of food to PLHIV and the introduction of an ARV injection that is administered once in 6 months. Participants also suggested the construction of more consultation rooms at clinics, strengthening of health information systems, decentralisation of ART services from clinics, delivery of chronic medicines and providing health education on ART at churches and workplaces. Community-based ART groups can address many of the issues raised by participants such as long waiting times and high transportation costs. Bernays et al. (2021) used a programmatic data analysis method from the Broad Reach International programme of 217 facilities in five districts of South Africa between 2016 and 2017 and revealed a rapid uptake of differentiated models of care (facility and out-of-facility-based). Approximately, 75% of eligible clients accepted the models, and there was a 10% increase in clients moving to community-based models (Bernays et al. 2021). It is unclear, though, if patients in this investigation were given the option to choose between the models and the standard of care.

### Strengths and limitations

The study’s strength lies in its unique research design, which prevented it from being constructed using pre-published information. From the gathered data, themes were objectively retrieved, and they were then contrasted with earlier conclusions drawn from the examined literature. The participants’ native tongue, isiXhosa, was used for the interviews. This is advantageous because it allows participants to express themselves completely. The interviews were then translated into English by the researcher, who is fluent in both isiXhosa and English. This suggests that the researcher interpreted the information while taking into account nonverbal clues and paying attention to what was said and how it was spoken. Consequently, the subliminal messages that were apparent in the participants’ statements were assimilated.

There were some limitations to the study despite the researcher’s best efforts to guarantee the accuracy of the results. Firstly, the study’s non-probability purposive sampling technique might have exacerbated selection bias. Secondly, because of the inherent bias in face-to-face interviews, some participants might give responses that are acceptable in society. Thirdly, some participants expressed discomfort with audio recording because they felt anxious using the devices, which would have a negative impact on their responses. Fourthly, the researcher had to wait a long period for a private room because they were not always available. Fifthly, some participants were unable to wait because they had to rush back to finish other household duties or go back to work after their work shifts finished.

### Recommendations

From the study findings, several recommendations have been made:

- Including farming communities holistically in combined methods to fight HIV, AIDS, TB and STIs is crucial. Therefore, an HIV prevention plan ought to make use of events such as the International Day against Drug Abuse and Illicit to inform farming communities about the link between drug abuse, STIs, HIV and ART adherence.
- Together with the private sector, the government can make it possible for all PLHIV taking ART – including those living in isolated rural communities – to receive crucial information so they can comprehend and put it to use.
- A new community-based ART delivery approach must involve the clinic committee, the ward council member and many community debates. To lessen the difficulties, farming communities can operationalise the Centralised Chronic Medicines Dispensing Distribution (CCMDD) model, which provides PLHIV with a 6-month supply of ART without requiring them to see a clinician until there are issues. Interventions aimed at lowering stigma and discrimination ought to focus on the community, workplace and family settings.
- Treatment interventions and family-based testing should be part of the ART plan. This would make it easier to educate family members about HIV and encourage them to seek and adhere to treatment.
- It is necessary to implement a filling system in accordance with the District Health Information System (DHIS) recommendations. All clinic records should be completed with appointment dates and clinic file numbers for convenience of access. It should be the responsibility of a committed worker to fill out, record and gather clinic data.
- To enhance the spread of knowledge about HIV, AIDS and ART, radio communication, social media for health information and healthcare clinic conversations should be reinforced.
- Healthcare professionals must receive compassionate training to suit the specific needs of PLHIV in order to assist them adhere to ART. It is essential to encourage pleasant, non-judgemental and positive attitudes. Prior research has also suggested that educating medical staff on ethical care practices can help to increase adherence.
- Lastly, employers should show their support by offering wellness programmes on their farms, enabling all workers to participate without worrying about the no-work, no-pay clause. This would ensure that all workers have equitable access to information regarding HIV/AIDS, ART and other chronic illnesses including diabetes and hypertension.

### Recommendations for further research

The findings described in this study have been observed to both facilitate and restrict access to ART in rural areas in the OR Tambo District Municipality farming community. To facilitate the generalisation of the findings to other farming communities in South Africa, it is recommended that further research be conducted in other regions of the nation employing a quantitative approach.

## Conclusion

This study demonstrated that accessing ART is not simple in farming communities. Many factors play a role in the process, which ultimately leads to the farming communities’ inability to access and adhere to ART. Farming communities found that obstacles to receiving ART were the difficulty in getting to medical facilities because of long distances and lack of transportation funds or transport. This study also revealed that, when it comes to providing ART and providing adherence support for clients on ART in farming communities, there is still a significant gap in the healthcare services available. Following the study’s objectives, the obstacles to PLHIV access to ART were identified in farming communities and practical suggestions were made for enhancing that access in OR Tambo District Municipality farming communities. Such barriers include having to travel long distances to reach the clinic for ART, and there are at times referrals made to other clinics because of stock outs. This means there are possibilities of returning home without ART. Stigma and discrimination, religious and traditional beliefs and socio-economic factors such as transport costs were also identified as barriers. Healthcare system barriers to ART access included the unavailability of an adequate supply of drugs and healthcare staff. Even though the obstacles to receiving ART were made public, the participant responses revealed that they are aware of the distinction between HIV and AIDS, understanding that the former is a virus and the latter is a disease. This demonstrated that most people knew the distinction between HIV and AIDS. The researcher assumed that when participants knew the distinction between HIV and AIDS, they would understand the significance of adhering to treatment and would seek help if they tested positive for HIV or had already tested positive for HIV but were not yet on ART. The study also showed that while most participants knew about ART, their knowledge of the many kinds of ART was restricted. The lack of access to ART in farming communities is influenced by a variety of complex and dynamic factors, including socioeconomic, health system and individual factors. According to the study’s findings, low socioeconomic level, limited social support and limited socio-cultural standing are all associated with inadequate access to ART in rural areas. Thus, to improve ART adherence and access and thereby extend the life expectancy of PLHIV in farming communities, HIV initiatives and interventions must prioritise addressing these variables.
